# Impact of Multicohort Human Papillomavirus Vaccination on Cervical Cancer in Women Below 30 Years of Age: Lessons Learned From the Scandinavian Countries

**DOI:** 10.1093/infdis/jiae584

**Published:** 2024-11-21

**Authors:** Ståle Nygård, Thea Eline Hetland Falkenthal, Tina Sture, Elsebeth Lynge, Miriam Elfström, Mari Nygård

**Affiliations:** Department of Research, Cancer Registry of Norway, Norwegian Institute of Public Health, Oslo, Norway; Department of Research, Cancer Registry of Norway, Norwegian Institute of Public Health, Oslo, Norway; Department of Research, Cancer Registry of Norway, Norwegian Institute of Public Health, Oslo, Norway; Zealand University Hospital, Nykøbing Falster, Denmark; Center of Cervical Cancer Elimination, Department of Clinical Science Intervention and Technology, Karolinska Institutet, Stockholm, Sweden; Department of Research, Cancer Registry of Norway, Norwegian Institute of Public Health, Oslo, Norway

**Keywords:** human papillomavirus (HPV), single-cohort vaccination, multicohort vaccination, Scandinavian countries, cervical cancer incidence rates

## Abstract

In Scandinavia, human papillomavirus (HPV) vaccination programs started in 2007–2008 in Sweden and Denmark with HPV vaccination offered to multiple cohorts of young girls, while in Norway this was offered to a single cohort only. Interestingly, in Sweden and Denmark, cervical cancer incidence in young women decreased markedly from 2017 to 2018, while in Norway a steady increase was seen until 2020. As the 3 countries are very similar in other factors important for cervical cancer incidence rates, like cervical cancer screening, the observed difference is most likely due to differences in the multicohort vaccination.

## Teaser

Recently, cervical cancer incidences have decreased markedly in young women in the Scandinavian countries. The decrease in Denmark and Sweden was seen many years earlier than in Norway, most likely because of earlier multicohort HPV vaccination in Denmark and Sweden.

While cervical cancer is preventable and curable when detected early, it remains the fourth most common form of cancer among women worldwide. The World Health Organization announced in 2018 a call to action to eliminate cervical cancer as a public health problem, defined as fewer than 4 new cases per 100 000 women per year [1]. To achieve this goal a so-called 90-70-90 target was formulated: vaccination, 90% of girls to be fully vaccinated with a human papillomavirus (HPV) vaccine by the age of 15 years; screening, 70% of women to be screened using a high-performance test by the age of 35 years, and again by the age of 45 years; and treatment, 90% of women with precancer treated and 90% of women with invasive cancer managed.

Cervical cancer screening, that is testing asymptomatic women for presence of precancerous cervical lesions followed by treatment of the lesions before they progress to invasive disease, has played an important role in reducing the incidence of cervical cancer in many countries. In the Nordic countries, screening is estimated to have reduced the incidence of cervical cancer 3-fold after it was introduced in the second half of the last century [2]. From 2000, the reduction in cervical cancer incidence leveled off in Scandinavia, indicating that the traditional cytology-based screening by then had reached most of its potential for further reduction of cancer incidences.

**Figure 1.**
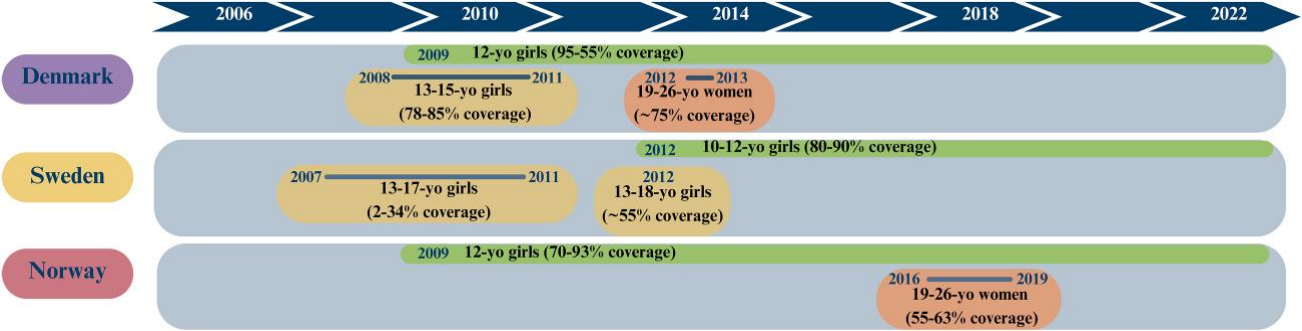
Female HPV vaccination programs in Denmark, Sweden, and Norway 1943–2020. Single-cohort programs are indicated by green color, and multicohort programs below sexual debut age by yellow and above sexual debut age by orange color.

In 2009, Denmark and Norway implemented routine HPV vaccination of 12-year-old girls as part of the childhood vaccination programs, in Denmark organized with vaccination by general practitioners and in Norway school based (Figure 1). Sweden started school-based HPV vaccination of 10–12-year-old girls in 2012. The coverage has been high in all the 3 countries: in Norway, 93% received at least 1 dose as part of the program in 2019, compared to 86% in Sweden and 79% in Denmark [3]. In Sweden and Denmark, but not in Norway, catch-up programs were started almost at the same time as the initiation of the childhood vaccination program (Figure 1).

In Denmark, fully subsidized catch-up vaccination of 13–15-year-old girls (born 1993–1995) was available from 2008. The coverage was 78%–85%. A catch-up vaccination for women aged 19–26 years (born 1985–1992) was offered in 2012–2013. The coverage was 75%. In addition, some girls and young women paid for the vaccine themselves and were vaccinated before the program started, up to 21 000 by the start of 2008 [4].

In Sweden, partially subsidized catch-up vaccination of 13–17-year-old girls was offered from 2007, and fully subsidized vaccination for the same age group from 2012. The partially subsidized vaccination coverage was low, ranging from 2% in 2007 to 34% in 2011. The coverage of the subsidized vaccination was higher, with an average of 55% [5].

In Norway, a catch-up vaccination program with the bivalent vaccine in a 3-dose schedule was effective from November 2016 to June 2019, targeting women born in 1991 and later who had not been previously vaccinated in the routine program, that is women 19–28 years of age. The vaccination was offered free of charge through primary care services. The coverage was 55%–63% (http://khs.fhi.no).

## METHODS

The age group of 25–29-year-old women is the youngest group with a considerable cervical cancer incidence rate, and thus this is the age group where we first expect to see any effect of the HPV vaccination programs (Supplementary Figure 1). For this age group, we retrieved cervical cancer incidences for Norway, Sweden, and Denmark for the period 2000–2021 from Nordcan 2.0 [6] and for 2022 from the 3 countries’ national cancer registries. Joinpoint regression analyses, assuming exponential growth on segments, were performed according to Kim et al [7]. The optimal number of joinpoints for each country was decided by permutation tests. An annual percentage change (APC) was calculated for each segment restricted by the joinpoints.

## RESULTS

**Figure 2.**
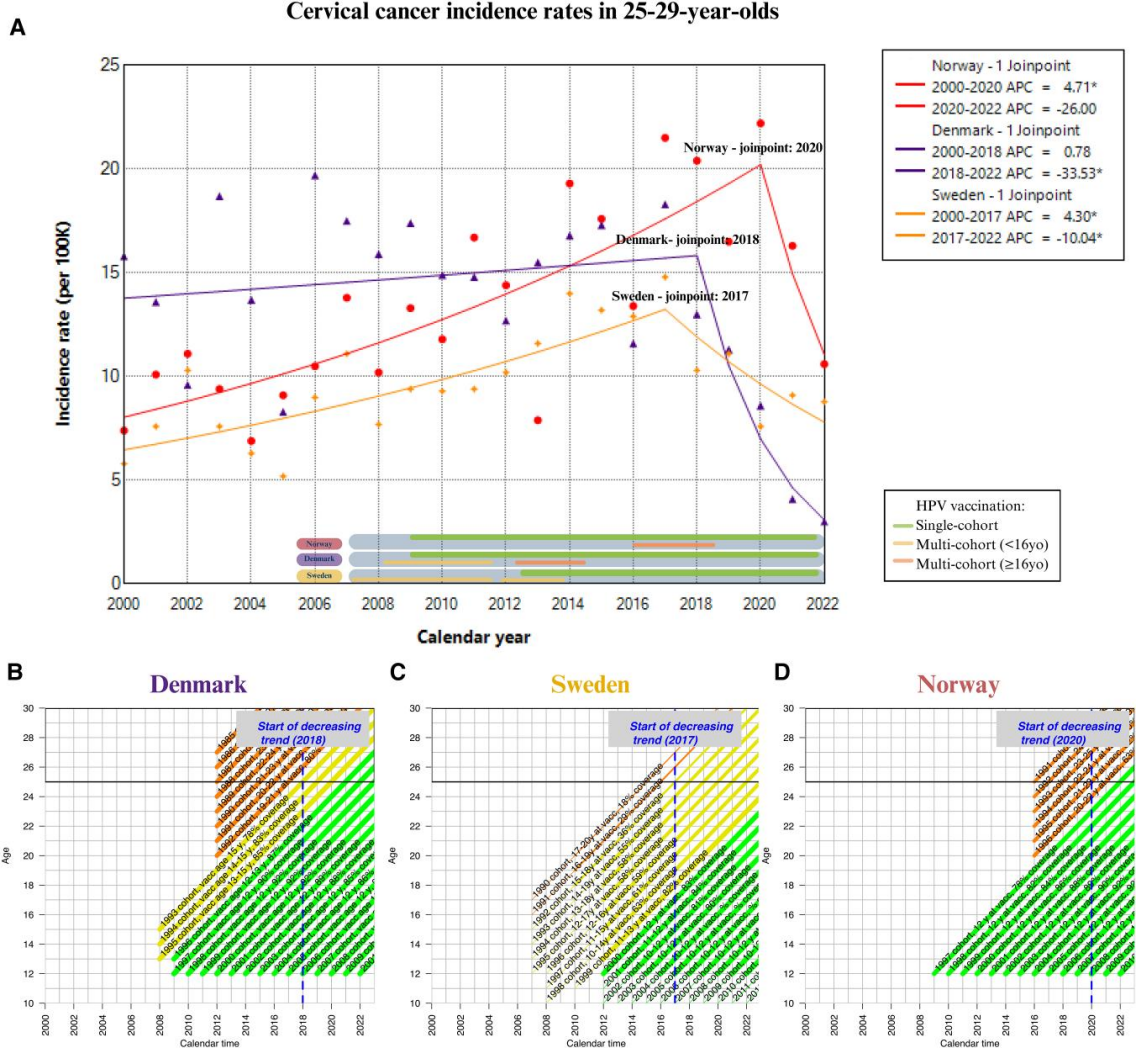
*A,* Cervical cancer incidence rates in 25–29 year olds, 2006–2022 in Denmark, Sweden, and Norway. The points are observed annual incidence rates and the lines are estimated using joinpoint regression. *B*–*D,* Lexis diagrams of birth cohorts offered HPV vaccination in (*B*) Denmark, (*C*) Sweden, and (*D*) Norway. The thickness of the lines is proportional to the vaccination coverage. The age group 25–29 years is indicated by black horizontal lines. Blue dotted vertical lines indicate the start of declining incidence rates based on joinpoint regression analysis. Single-cohort vaccination is indicated with green color, and multicohort with yellow (before median sexual debut age) and orange color (after median sexual debut age). *Significantly different from 0. Abbreviations: APC, annual percentage change; HPV, human papillomavirus; vacc, vaccination.

For all the 3 countries, the joinpoint regression resulted in 1 joinpoint, representing a change from increasing to decreasing incidences (Figure 2*A*). In Sweden, a significant decrease was seen from 2017 (APC = −10; 95% confidence interval [CI], −28 to −1). In Denmark, a steeper decrease was seen from 2018 (APC = −33; 95% CI, −50 to −21). In Norway, the decline in cervical cancer incidences was not seen in the young women before 2021; although steep, the decline was not statistically significant (APC = −26; 95% CI, −46 to 3).

## DISCUSSION

In the randomized controlled trials of the HPV vaccines, the protective effect against high-grade cervical lesions was seen in women who were HPV-naive at the time of vaccination [8, 9]. Vaccination has therefore been targeted at girls below the age of sexual debut, in many countries at age 12. The protective effect of vaccination on cervical cancer incidence will therefore show up only several years after vaccination, and the effect is expected to be visible first in the age group 25–29 years.

In Sweden, girls HPV vaccinated at age 15 years reached age 25 years in 2017 and in Denmark in 2018 (Figure 2*B* and 2*C*). This pattern was reflected in a decrease in cervical cancer incidence in Sweden and Denmark from these years onwards (Figure 2*A*). The decrease in incidence was stronger in Denmark, where the vaccination coverage of the early multicohort vaccination was much higher than in Sweden (78%–85% vs 2%–24%). In the beginning of the Norwegian vaccination program only 12-year-old girls were targeted, and this birth cohort reached the age of 25 years only in 2022 (Figure 2*D*).

Screening attendance and methodology should also be considered when looking at cervical cancer incidence trends. Like HPV vaccination, screening reduces the cancer incidence on later ages, as a treated precancerous lesion now, is a reduced risk of cancer at an older age. Thus, improvements in screening at ages 25–29 years may reduce cancer incidence only after age 30. In fact, improving sensitivity of screening of 25–29-year-old women may *increase* cervical cancer incidence for that age groups, as more nonsymptomatic early-stage cancer cases are detected. A recent study in Denmark found increased incidence rates after HPV-based screening was implemented in the screening program [10].

Liquid-based cytology (LBC) has been the main screening methodology for 25–29-year-olds between 2010 and 2022. Norway implemented LBC screening from 2012 to 2014, some years later than in Denmark (2002–2014) and Sweden (2010). SurePath LBC provides a better protection against cervical cancer than conventional cytology [11], but the rapid decrease in cervical cancer incidence seen after the childhood HPV-vaccinated cohorts entered the age group 25–29 years is unlikely to be attributable to the use of LBC. HPV-based screening provides a better protection than cytology [12], but HPV testing was not used in women below 30 years, except in Stockholm starting in 2019. Screening attendance was fairly stable in the study period, apart from some decrease during coronavirus disease 2019 (COVID-19) [13].

Cross-sectional surveys have shown very similar sexual behavior patterns in the Scandinavian countries. The median sexual debut age for birth cohorts 1989–1994 (22–27 years of age in 2016) were 16 years for all the 3 countries, and median number of lifetime partners for 25–29-year-old women were 6 in Norway and Denmark, and 8 in Sweden [14]. The HPV distribution was similar between the 3 countries in the prevaccination era, with percentages of 18–26-year-old women being positive to high-risk HPV of 49.6% in Norway, 37.5% in Sweden, and 43.6%, in Denmark [15].

As we have discussed, the Scandinavian countries are very similar in terms of sexual behavioral patterns, prevaccination exposure to high-risk HPV, screening methodology, and start of childhood vaccination. The difference between the Scandinavian countries in time trends in cervical cancer incidence among 25–29-year-old women therefore strongly suggests that the multicohort implementation of HPV vaccination in young girls in Sweden and Denmark resulted in an earlier decrease in cervical cancer incidence than the single-cohort implementation in Norway. In general, one should be cautious about inferring a causal relationship from an observed statistical association. Unmeasured confounders, that is factors affecting both the exposure (here vaccination) and the outcome (here cancer), could have contributed to the association. Potential confounders in our setting are socioeconomic status, education level, general health literacy, and more specifically HPV awareness, that can impact both vaccine coverage and cancer incidences, the latter most likely mediated through screening attendance and sexual behavior. As we compare entire age groups, and not population subgroups, confounding is relevant only if there were changes in the age-group 25–29 years during an about 10-year time period up to 2022. However, we are not aware of any major changes in any of the confounding variables mentioned during the study period.

Many countries are yet to implement national HPV vaccination programs. Based on the experience from the Scandinavian countries the take-home message is clear: By starting off offering vaccination to multiple cohorts below sexual debut age, and not restricting the vaccination to single cohorts of 10–12-year-olds, the time to cervical cancer elimination may be reduced by several years.

## Supplementary Data

Supplementary materials are available at *The Journal of Infectious Diseases* online (http://jid.oxfordjournals.org/). Supplementary materials consist of data provided by the author that are published to benefit the reader. The posted materials are not copyedited. The contents of all supplementary data are the sole responsibility of the authors. Questions or messages regarding errors should be addressed to the author.

## Supplementary Material

jiae584_Supplementary_Data

## Note

***Financial support.*** No financial support was received for this work.
