# Supplementary material for: Impact of Multicohort Human Papillomavirus Vaccination on Cervical Cancer in Women Below 30 Years of Age: Lessons Learned From the Scandinavian Countries
Source: J Infect Dis. 2024 Nov 21;231(3):e497–500. doi: 10.1093/infdis/jiae584 (PMC11911778; doi:10.1093/infdis/jiae584)
Supplement: jiae584_Supplementary_Data [file jiae584_supplementary_data.zip › supp fig legend.docx]

Supplementary figure 1: Age-specific cervical cancer incidence rates (IR) in Norway 2000-2006. The age group 25-29 years is the youngest with a considerable IR and is marked with black color.
