# Supplementary figures and images for: Impact of Multicohort Human Papillomavirus Vaccination on Cervical Cancer in Women Below 30 Years of Age: Lessons Learned From the Scandinavian Countries
Source: J Infect Dis. 2024 Nov 21;231(3):e497–500. doi: 10.1093/infdis/jiae584 (PMC11911778; doi:10.1093/infdis/jiae584)

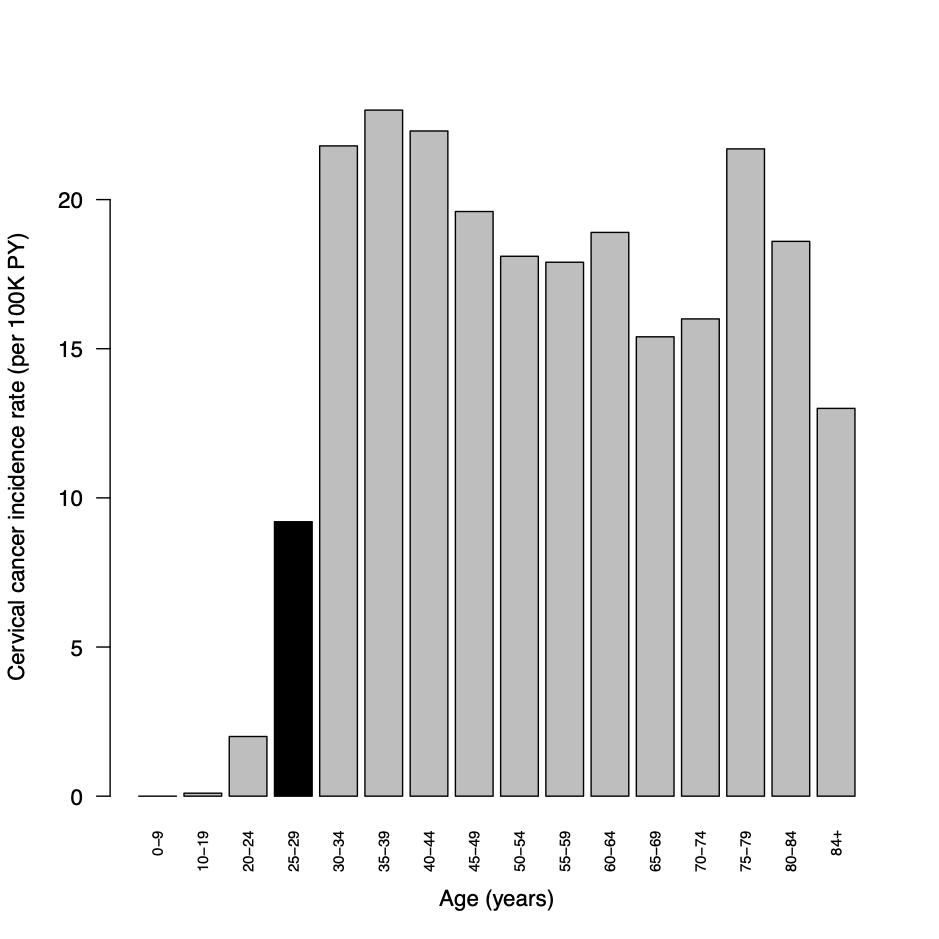

Supplement: jiae584_Supplementary_Data [file jiae584_supplementary_data.zip › sup-fig-1.png]
